# Optimizing cardiac surgery outcomes in morbid obesity: Surgical, anesthetic, perfusion, and critical care considerations, a narrative review

**DOI:** 10.1051/ject/2026021

**Published:** 2026-09-15

**Authors:** Salman Pervaiz Butt, Salman Abdulaziz, Nabeel Razzaq, Rhea Anand, Nilesh Srivatav, Yasir Saleem, Olga Lender, Mairead Griffin, Azreen Afzal, Vivek Kakar, Fazil Ashiq, Arun Kumar, Arshad Ghori, Jehad Ramahi, Mitesh Badiwala, Umer Darr, Gopal Bhatnagar

**Affiliations:** 1 Perfusion Services, Heart Vascular and Thoracic Institute, Cleveland Clinic Abu Dhabi United Arab Emirates; 2 Department of Critical Care, King Fahad Medical City Riyadh Saudi Arabia; 3 Western University London Ontario Canada; 4 Cardiac Surgery, Heart Vascular and Thoracic Institute, Cleveland Clinic Abu Dhabi United Arab Emirates; 5 Perfusion Department, Cardiac Surgery, All India Institute of Medical Sciences New Delhi India; 6 Mid Cheshire Hospitals NHS Foundation Trust UK; 7 Critical Care Institute, Cleveland Clinic Abu Dhabi United Arab Emirates; 8 Cardiothoracic Anaesthesia, Anesthesiology Institute, Cleveland Clinic Abu Dhabi United Arab Emirates

**Keywords:** Morbid obesity, Cardiac surgery, Cardiopulmonary bypass

## Abstract

*Introduction*: The global prevalence of morbid obesity is increasing, presenting challenges in cardiac surgery. Morbid obesity (body mass index ≥40 kg/m^2^) is associated with a high burden of cardiometabolic comorbidities and obesity-specific physiological alterations that increase perioperative risk and complicate surgical, anesthetic, and cardiopulmonary bypass management. As the number of obese patients presenting for cardiac surgery rises, a synthesis of current evidence and perioperative strategies is needed to optimize outcomes in this high-risk population. *Methods*: This narrative review identified relevant literature through targeted searches of PubMed and Google Scholar, supplemented by screening the reference lists of key articles. Search terms included combinations of “cardiac surgery,” “morbid obesity,” “obesity,” “cardiopulmonary bypass,” “anesthesia,” “perfusion,” “postoperative complications,” and “critical care.” Studies were mapped to perioperative domains, including anesthesia and respiratory management, surgical and wound-related considerations, perfusion and cardiopulmonary bypass strategy, intensive care unit (ICU) outcomes, renal complications, atrial fibrillation, bleeding, thromboembolism, and mortality. The evidence base was predominantly observational and review-based, with limited randomized evidence. *Discussion*: Obesity poses multifactorial challenges in cardiovascular surgery by altering cardiac morphology, hemodynamics, and perioperative outcomes. Associated comorbidities such as diabetes, obstructive sleep apnea, and pulmonary hypertension increase operative risk and complicate anesthetic and perfusion management. Obese patients demonstrate altered pharmacokinetics, reduced pulmonary compliance, and heightened inflammation, requiring individualized strategies. Available evidence suggests that lean body mass-informed perfusion, selected off-pump CABG, and early extubation may reduce complications, although high-quality randomized evidence remains limited. *Conclusion*: Cardiac surgery in morbidly obese patients requires a coordinated, physiology-driven perioperative strategy to address obesity-specific anatomical and functional challenges. Tailored anesthetic, perfusion, and surgical approaches, supported by close multidisciplinary collaboration, are central to optimizing outcomes. Further research is needed to refine perioperative protocols, improve risk stratification beyond BMI, and clarify long-term cardiovascular outcomes following surgery in this population.

## Introduction

The prevalence of morbid obesity continues to rise globally, with significant implications for patients undergoing cardiac surgery. Defined as a body mass index (BMI) ≥40 kg/m^2^, morbid obesity is associated with a high burden of cardiovascular and metabolic comorbidities, including diabetes mellitus, obstructive sleep apnea, pulmonary hypertension, and chronic kidney disease. These conditions compound perioperative risk and contribute to higher rates of respiratory failure, acute kidney injury, wound complications, and prolonged ICU stay following cardiac surgery [1].

In addition to comorbidity burden, obesity introduces unique physiological and technical challenges that affect surgical exposure, anesthetic management, cardiopulmonary bypass strategies, and postoperative care. Altered pharmacokinetics, reduced pulmonary compliance, increased systemic inflammation, and difficulties with vascular access and wound healing necessitate individualized, multidisciplinary perioperative management. As the proportion of patients with elevated BMI presenting for cardiac surgery continues to increase, there is a growing need to synthesize available evidence and highlight practical strategies to optimize outcomes in this high-risk population [2–5]. Optimizing outcomes requires coordinated surgical, anesthetic, perfusion, nursing, and ICU strategies across the perioperative pathway.

## Methods

This narrative review identified relevant literature through targeted searches of PubMed and Google Scholar, supplemented by screening the reference lists of key articles. Search terms included combinations of “cardiac surgery,” “morbid obesity,” “obesity,” “cardiopulmonary bypass,” “anesthesia,” “perfusion,” “postoperative complications,” and “critical care.” Articles were selected according to their relevance to surgical, anesthetic, perfusion, nursing, and postoperative management of adult cardiac surgical patients with obesity.

Studies were mapped to perioperative domains, including anesthesia and respiratory management, surgical and wound-related considerations, perfusion and cardiopulmonary bypass strategy, ICU outcomes, renal complications, atrial fibrillation, bleeding, thromboembolism, and mortality. The evidence base was predominantly observational, review-based, psychological, and expert/practice-based, with limited randomized evidence. No quantitative pooling or formal risk of bias assessment was performed. Findings were synthesized thematically to identify clinically relevant patterns, areas of uncertainty, conflicting evidence, and practical perioperative implications. A total of 56 references were included and mapped thematically across perioperative domains, including cardiovascular risk, surgical and wound considerations, anesthesia and respiratory management, perfusion and CPB strategy, anticoagulation, ICU outcomes, renal complications, atrial fibrillation, bleeding and transfusion, thromboembolism, and critical care management.

## Discussion

### Obesity and cardiovascular surgery

The rising prevalence of obesity has important implications for cardiovascular surgery because obesity does not act as an isolated risk factor, but as a modifier of cardiometabolic disease, operative complexity, and postoperative recovery. Existing knowledge has established that obesity promotes cardiovascular disease through dyslipidemia, type 2 diabetes, hypertension, sleep disordered breathing, systemic inflammation, insulin resistance, and activation of the renin–angiotensin–aldosterone and sympathetic nervous systems. Visceral, pericardial, and epicardial adiposity further contribute to coronary microvascular dysfunction, atherosclerosis, and increased carotid intima media thickness, all of which may affect perioperative risk assessment and intraoperative perfusion planning. Obesity related comorbidities such as diabetes, sleep apnea, hypoventilation syndrome, and pulmonary hypertension identify patients whose risk is driven more by physiological reserve than BMI alone. Notably, each 5 kg/m^2^ increase in BMI has been associated with a 16% increase in sudden cardiac death risk, reinforcing the cardiovascular relevance of obesity beyond surgical technical difficulty [1].

Obesity also produces structural and electrophysiological cardiac changes that are directly relevant to cardiac surgery. Myocardial fat infiltration and fibrosis contribute to LV diastolic dysfunction and heart failure with preserved ejection fraction, while LV hypertrophy, RV dilatation, and RV dysfunction may reduce perioperative tolerance to volume shifts, hypoxia, and pulmonary hypertension. Similarly, atrial remodeling, intramyocardial adiposity, and epicardial fat-related fibrosis increase susceptibility to AF, with each 5 kg/m^2^ increase in BMI associated with an approximately 29% higher risk of incident AF. The implication is that obesity should not be assessed solely as body size, but rather as a disease state associated with altered ventricular function, increased pulmonary vascular load, elevated arrhythmia risk, and reduced physiological reserve [1].

There is increasing focus on whether obesity related risk can be modified before surgery and whether perioperative strategies should differ by obesity phenotype. Lifestyle interventions and weight loss can improve components of metabolic syndrome, although their impact on coronary artery disease event rates remains less certain. Bariatric surgery may reduce coronary artery disease risk and improve outcomes in heart failure and AF, but its role before cardiac surgery is limited by clinical urgency and lack of direct perioperative evidence. GLP-1 receptor agonist-based therapies such as semaglutide represent an emerging approach to weight reduction in adults with overweight or obesity; however, their direct impact on cardiac surgical outcomes remains unproven and should be considered hypothesis-generating rather than established perioperative practice. Potential perioperative concerns include delayed gastric emptying, gastrointestinal intolerance, possible aspiration risk, treatment discontinuation with weight regain, and loss of lean or bone mass. Therefore, GLP-1 receptor agonists should be framed as a potential tool for cardiometabolic optimization rather than an established cardiac surgical risk-reduction strategy [6, 7].

The perioperative literature has also moved from simply describing obesity-associated risk to identifying potentially modifiable strategies. Djouani et al. identified preoperative respiratory muscle training, off-pump CABG, and early extubation as possible approaches to reduce perioperative morbidity in morbidly obese patients [8]. These strategies are biologically plausible because they target mechanisms central to obesity related risk, including reduced respiratory reserve, prolonged ventilation, and CPB-associated inflammatory or renal injury. However, the evidence remains limited, and these interventions should be interpreted as pragmatic risk-reduction strategies rather than definitive standards of care, pending further randomized evaluation.

Preoperative optimization is therefore best viewed as targeted physiological risk stratification rather than generic clearance. Benalcazar et al. emphasized thorough cardiovascular evaluation, including history, examination, and diagnostic assessment, particularly in patients requiring CABG or valve procedures [9]. In obese cardiac surgical patients, this assessment should specifically identify sleep-disordered breathing, pulmonary hypertension, renal vulnerability, airway difficulty, and functional limitation, as these factors are more actionable than BMI alone. Anesthetic planning, hemodynamic monitoring, fluid strategy, postoperative mobilization, and respiratory support should therefore be integrated before the day of surgery rather than managed reactively.

The relationship between obesity and postoperative outcomes is inconsistent across studies, which is central to interpreting the literature. Kumar et al. found that BMI >40 kg/m^2^ independently predicted acute kidney injury after cardiopulmonary bypass, supporting the view that severe obesity increases vulnerability to renal injury [10]. In contrast, Pullan et al. reported lower in-hospital mortality among obese patients undergoing off-pump CABG, while Song et al. found that obesity did not worsen perioperative or long-term survival after off-pump CABG and that underweight patients had worse outcomes [11, 12]. Conversely, Jiang et al., using the MIMIC-III database, found obesity was associated with higher 28-day postoperative mortality, partly mediated by prolonged ventilation [13]. These conflicting findings suggest that obesity is not uniformly harmful or protective; instead, outcomes likely depend on obesity severity, comorbidity burden, procedure type, CPB exposure, respiratory complications, and institutional expertise.

Overall, the existing evidence confirms that obesity alters cardiovascular structure, respiratory reserve, renal risk, and perioperative physiology, while newer evidence increasingly focuses on individualized strategies such as off-pump CABG, early extubation, respiratory prehabilitation, and more detailed preoperative risk assessment. However, the benefit of these approaches is not uniform. Off-pump CABG, for example, may be advantageous in selected obese patients by avoiding CPB-related morbidity, but outcomes are likely influenced by surgeon and institutional experience; high volume off-pump centers appear to achieve better results than centers where the technique is used less frequently [14]. Therefore, the most defensible conclusion is not that obesity alone should dictate a specific operative strategy, but that morbid obesity should trigger a multidisciplinary, physiology-driven pathway that accounts for respiratory reserve, renal vulnerability, cardiac remodeling, technical complexity, and local procedural expertise.

### Surgical considerations

Cardiac surgery in morbidly obese patients is technically challenging, but surgical risk is not explained by body size alone. Rather, obesity creates a sequence of linked operative hazards: increased tissue depth limits exposure, limited exposure prolongs operative and cardiopulmonary bypass time, and prolonged tissue handling may increase bleeding, wound complications, and postoperative recovery burden. Existing evidence shows that excess adipose tissue can impair access to the surgical field, requiring deeper retractors, longer instruments, modified positioning, and additional assistance to achieve adequate visualization. These technical constraints may increase operative duration and contribute to intraoperative blood loss, infection risk, and cardiopulmonary compromise related to prolonged anesthesia and bypass exposure [5, 8].

Surgical complications in morbid obesity should therefore be viewed as interrelated rather than isolated events. Increased subcutaneous tissue depth and altered microvascular perfusion may impair wound healing and increase the risk of surgical site infection, while the vascularity of adipose tissue can make hemostasis more difficult. These risks likely share overlapping mechanisms, including impaired tissue perfusion, increased tissue depth and dead space, greater tissue trauma, longer operative time, and challenges with glycemic and infection control. Consequently, surgical mitigation should focus on preoperative equipment planning, exposure strategy, meticulous hemostasis, dead space reduction, appropriate antibiotic dosing and redosing, and closure techniques that reduce sternal and soft tissue tension [5, 8, 15–18].

The feasibility of minimally invasive or alternative surgical approaches in morbid obesity remains uncertain and should be individualized rather than determined by BMI alone. In some patients, obesity may reduce the practicality of minimally invasive access because of tissue depth, restricted working space, and difficulty with peripheral cannulation. Conversely, avoiding larger incisions may theoretically reduce wound morbidity in selected patients when performed by experienced teams. This tension highlights the need to align the surgical approach with patient anatomy, procedure complexity, cannulation feasibility, and institutional expertise.

Positioning and vascular access represent additional areas where obesity modifies surgical risk. Restricted mobility, operating table limitations, and difficult transfers increase the risk of pressure injuries, nerve injuries, staff injuries, and delays in operative workflow. Increased subcutaneous tissue depth may also complicate vascular access for cardiopulmonary bypass cannulation, particularly when peripheral or femoral access is required. In these cases, ultrasound guidance, modified access techniques, careful cannula positioning, and anticipation of cannula compression or kinking may reduce the risk of inadequate venous drainage or arterial inflow. Obesity related immobility and altered postoperative hemodynamics also increase susceptibility to venous stasis, deep vein thrombosis, and pulmonary embolism, reinforcing the need for early mobilization and thromboprophylaxis planning [5, 8, 15–17, 19–21].

Overall, existing literature establishes that morbid obesity increases surgical complexity through impaired exposure, wound vulnerability, access difficulty, and positioning risk. What is newer is the shift toward proactive surgical pathway planning, in which obesity is treated as a predictable modifier of operative logistics rather than an unexpected technical problem. The practical implication is that surgical teams should use a preoperative obesity-specific checklist addressing exposure, instruments, table capacity, positioning, cannulation plan, conduit harvesting strategy, wound prevention, and postoperative mobilization. This approach converts obesity from a broad risk label into a set of actionable surgical planning decisions.

### Integrated airway, respiratory, cardiovascular, and anesthetic considerations

Anesthetic management in morbidly obese patients undergoing cardiac surgery should be approached as an integrated problem of airway risk, respiratory reserve, cardiovascular vulnerability, pharmacokinetic variability, and postoperative recovery. Existing evidence establishes that excess adiposity around the neck, reduced neck mobility, and increased pharyngeal tissue bulk can narrow the upper airway and make laryngoscopy, intubation, and mask ventilation more difficult. However, the clinical risk is not limited to technical airway difficulty. Reduced functional residual capacity, ventilation perfusion mismatch, and increased oxygen consumption shorten the safe apnea time, meaning that failed or prolonged airway attempts can rapidly lead to hypoxemic events. Therefore, airway planning should include ramped positioning, optimized preoxygenation, availability of advanced airway devices, and consideration of apneic oxygenation or awake intubation in selected high-risk patients [2, 22, 23].

Respiratory management is another area where obesity specific physiology should directly influence intraoperative strategy. Increased intra-abdominal pressure and thoracic restriction reduce lung compliance, increase airway pressures, and promote basal atelectasis, thereby increasing susceptibility to intraoperative hypoxemia and postoperative pulmonary complications. The key management tension is that adequate PEEP and recruitment may improve oxygenation, but excessive PEEP or aggressive recruitment may reduce venous return and impair hemodynamics, particularly in patients with pulmonary hypertension, RV dysfunction, or limited preload reserve. Thus, ventilation should be individualized rather than protocolized, balancing oxygenation, driving pressure, lung recruitment, and cardiovascular tolerance [24–26].

Cardiovascular and pharmacological considerations are closely linked to this respiratory risk. Obesity is associated with hypertension, coronary artery disease, heart failure, pulmonary hypertension, and altered ventricular loading conditions, all of which may reduce tolerance to hypoxia, vasodilation, fluid shifts, and positive pressure ventilation. At the same time, increased volume of distribution for lipophilic drugs and variable clearance make total bodyweight dosing unreliable for many anesthetic agents. Existing practice, therefore, increasingly supports individualized titration using ideal, adjusted, or lean body weight where appropriate, combined with depth of anesthesia, neuromuscular, hemodynamic, and end-organ monitoring. Monitoring itself may be technically difficult because arterial, central venous, or neuraxial access can be limited by adipose tissue, reinforcing the need for early planning, ultrasound guidance, and equipment availability.

Postoperative respiratory risk should be anticipated before induction rather than managed only after failed extubation. STOP-Bang screening can help identify patients at risk for obstructive sleep apnea, while obesity hypoventilation, reduced pulmonary reserve, and residual anesthetic effect increase the likelihood of hypoventilation, airway obstruction, and reintubation. Nutritional deficiencies such as vitamin D, iron, and B12 deficiency may further impair recovery, wound healing, and immune function, although their direct effect on cardiac surgical outcomes remains less well defined. The practical implication is that extubation should be treated as a planned intervention, incorporating full reversal of neuromuscular blockade, head-up positioning, adequate analgesia without excessive respiratory depression, early CPAP or non-invasive ventilation where indicated, and coordinated handover to ICU and respiratory therapy teams [27, 28].

Overall, existing knowledge confirms that obesity increases airway difficulty, reduces respiratory reserve, alters drug handling, and increases postoperative pulmonary risk. What is newer is the shift from viewing obesity as a general anesthetic risk factor toward using phenotype-based planning: identifying the patient with difficult airway anatomy, OSA or hypoventilation, pulmonary hypertension, RV dysfunction, renal risk, or limited functional reserve. This approach helps reconcile why not all obese patients experience the same anesthetic risk and supports a more precise strategy in which induction, ventilation, drug dosing, monitoring, extubation, and postoperative respiratory support are individualized rather than determined by BMI alone [29, 30].

### Perfusion considerations

Perfusion management in morbid obesity requires more than scaling circuit components and pump flow to body size. Obesity introduces anatomical challenges, including difficult cannulation, impaired venous drainage, and potential cannula compression or kinking, particularly during femoral cannulation, where abdominal mass may externally restrict flow. Increased circulating blood volume and altered body habitus may require adjustments in reservoir management, suction strategy, circuit positioning, and venous drainage support. Myocardial protection may also require individualization, as increased myocardial mass, ventricular hypertrophy, and altered metabolic demand may make fixed cardioplegia dosing less reliable. Larger patient size can prolong cooling and rewarming and may increase oxygen consumption, but oxygenator selection should balance adequate gas exchange reserve against unnecessary circuit size and hemodilution [3].

The central unresolved issue is how best to determine pump flow in severe obesity. Conventional CPB practice commonly uses a pump flow of approximately 2.4 L·min^−1^·m^−2^ under normothermia [31]. However, this approach assumes that body surface area reflects metabolic need, which may be misleading when excess body mass is predominantly adipose tissue. In contrast, Condello et al. used goal-directed perfusion targeting DO_2_i ≥ 280 mL·min^−1^·m^−2^, adjusting flow according to hemoglobin concentration, temperature, and metabolic conditions rather than maintaining a fixed indexed flow. This is important because it shifts the target from body size to oxygen delivery. Their finding of lower intraoperative lactate peaks, with associated reductions in postoperative creatinine, ventilation time, and ICU stay, suggests that perfusion adequacy may be better judged by oxygen delivery and metabolic response than by fixed flow alone [32].

Evidence comparing BSA-based and lean mass approaches further supports this physiology-driven interpretation. Fixed BSA-indexed flow may produce relative overperfusion during hypothermia because metabolic demand falls as temperature decreases, while BSA remains unchanged. Lean body mass has therefore been proposed as a more relevant surrogate for systemic metabolic requirement than actual weight BSA, particularly in patients with elevated BMI [33, 34]. Santambrogio et al. similarly challenged actual weight BSA flow by comparing conventional BSA-indexed flow with ideal BSA-based flow; although mixed venous oxygen saturation and diuresis were similar, ideal BSA-based flow was associated with fewer perioperative complications and reduced transfusion requirements [35]. Taken together, these studies do not prove that one formula is universally superior, but they consistently question the assumption that actual body weight should drive CPB flow in obesity.

Blessing et al. provide a highly relevant perfusion-focused analysis, evaluating 2,816 cardiopulmonary bypass (CPB) cases to assess whether circuit components can be appropriately downsized in obese patients when indexed to lean body mass rather than conventional body surface area (BSA) or body mass index (BMI). Their findings demonstrate that, in patients with elevated BMI, adequate perfusion can be achieved with smaller oxygenators when flow is calculated according to lean body mass, with the additional benefit of higher hemoglobin nadirs and no associated increase in renal dysfunction. These data do not suggest that obese patients have reduced metabolic requirements; rather, they highlight that adipose tissue should not be considered metabolically equivalent to lean tissue when determining CPB flow and oxygenator sizing. Clinically, this supports a more physiologically guided approach to perfusion management, avoiding routine oversizing based on actual weight BSA, which may exacerbate hemodilution, while also ensuring that circuit capacity is sufficient in patients with genuinely increased flow or gas exchange demands [33].

**Figure 1:**
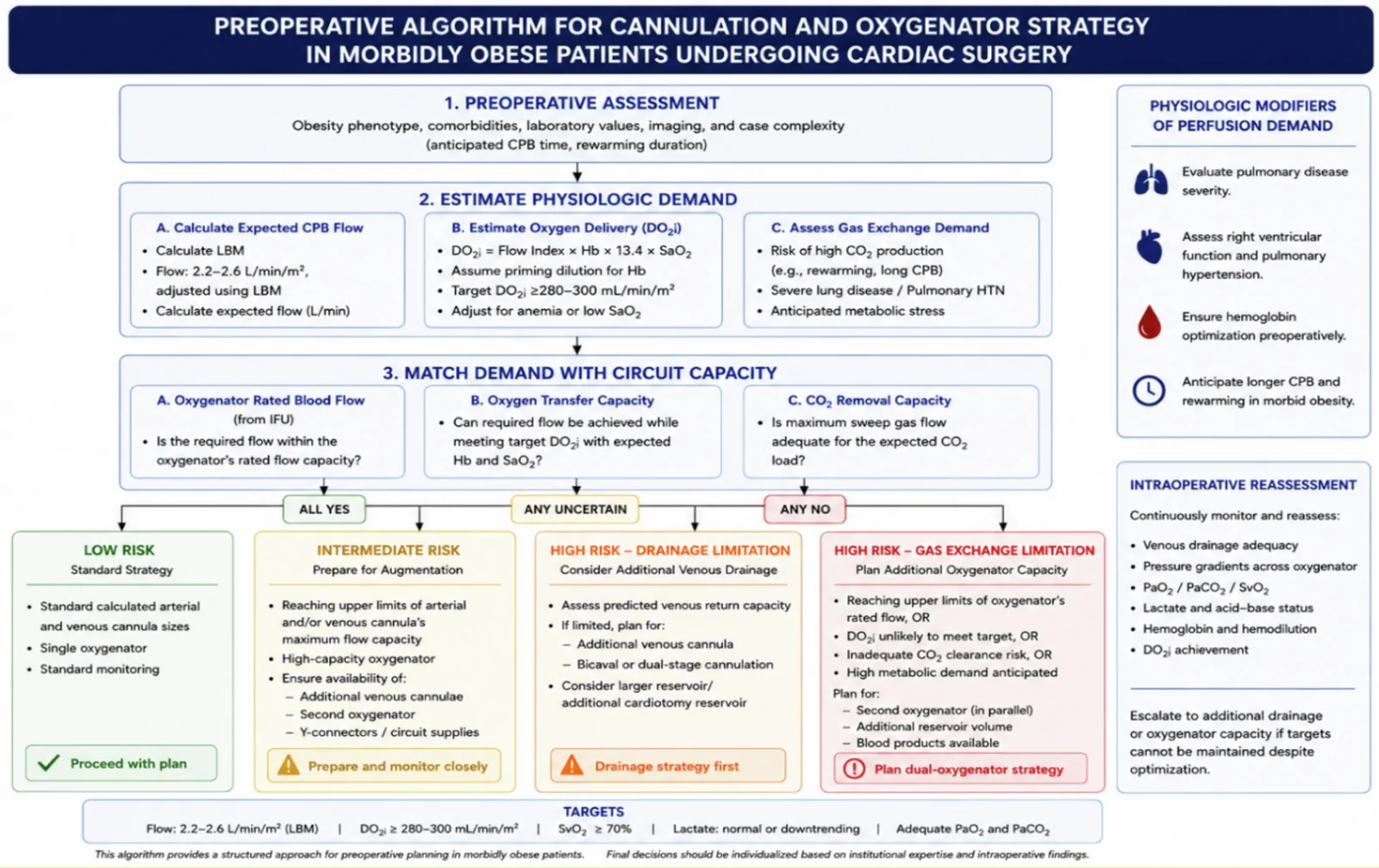
Preoperative algorithm for cannulation and oxygenator strategy in patients with morbid obesity undergoing cardiac surgery.

Oxygenator choice should therefore be individualized rather than determined by BMI alone. Most obese patients may be adequately supported with an oxygenator selected for expected lean flow requirements, oxygen delivery, hematocrit preservation, and gas exchange reserve. However, in extreme body size or unexpectedly high oxygen consumption, gas exchange may become the limiting factor. Hamilton et al. reported the use of a second oxygenator in a large patient, with SvO_2_ increasing from 38% to 60% after initiation of the second oxygenator. This case supports the feasibility of additional oxygenator capacity in selected patients, but it should be interpreted as a rescue or exceptional strategy rather than routine practice [20]. Because obesity may create either drainage limitation or gas-exchange limitation, Figure 1 proposes a preoperative algorithm for matching physiological demand to cannulation and oxygenator capacity.

The algorithm outlines a structured approach to estimating physiological demand, matching expected flow and gas-exchange requirements with circuit capacity, and identifying when additional venous drainage or oxygenator capacity should be prepared. Final decisions should be individualized according to device specifications, institutional expertise, and intraoperative findings.

Anticoagulation represents a parallel problem: total body weight may overestimate pharmacologically relevant dosing requirements in morbid obesity. Baker et al. reported reductions in heparin dose when lean body mass was used, while Aykut et al. found lean body- weight-based heparin dosing to be adequate in both obese and non-obese patients [36, 37]. These findings support an initial lean or adjusted body weight-based approach followed by ACT-guided titration, rather than fixed total body weight dosing. The same principle applies to protamine reversal, where excessive heparin dosing may lead to higher protamine exposure and potentially increase bleeding or adverse reactions. Therefore, anticoagulation in obese patients should be treated as a monitored titration strategy rather than a simple weight-based calculation.

Data specifically addressing temperature management in patients with obesity during cardiopulmonary bypass are limited. Available evidence suggests that microcirculatory perfusion is not significantly different between obese and non-obese patients during CPB, indicating that obesity does not independently impair microvascular response to hypothermia or rewarming [38]. However, obesity is associated with increased oxygen consumption, blood volume, and metabolic demand, which become particularly relevant during the rewarming phase. Metabolic demand may rise disproportionately during rewarming, while oxygen delivery may remain constrained by hemodilution and circuit factors. Consequently, the principal risk in patients with obesity is not temperature management per se, but a supply-demand mismatch during rewarming. Rapid rewarming and temperature overshoots should be avoided, and the metabolic demand should be anticipated. Flow, hemoglobin concentration, gas exchange, and oxygen delivery should be optimized before supply–demand mismatch develops.

Overall, current evidence challenges the use of actual-weight BSA as the sole determinant of perfusion strategy in morbid obesity. Although BSA-indexed flow remains common, lean body mass provides a more physiologically appropriate starting point, while intraoperative adequacy should ultimately be judged by DO_2_, hemoglobin concentration, lactate, venous saturation, temperature, renal risk, and end-organ perfusion. The available evidence remains limited and heterogeneous, particularly regarding optimal oxygenator sizing and anticoagulation; therefore, the most defensible approach is an individualized, goal-directed strategy that combines LBM-based estimation with dynamic DO_2_ monitoring, while avoiding underperfusion, unnecessary flow escalation, circuit oversizing, hemodilution, and excessive heparin/protamine exposure.

## Practical recommendations for perfusionists

Table 1 translates the above perfusion principles into practical intraoperative recommendations for flow strategy, oxygenator selection, anticoagulation, and temperature management.

**Table 1:** Practical recommendations for perfusion management in patients with obesity undergoing cardiopulmonary bypass.

Domain	Key recommendations	Rationale/evidence	Monitoring/targets
Flow strategy (LBM vs DO_2_i)	• Use LBW to estimate initial flow over actual-weight BSA.	• LBM correlates better with metabolic demand and allows safe circuit downsizing, improved hemoglobin, and reduced renal complications (Blessing et al. [33]).	DO_2_i > 280–300 mL/min/m^2^
	• Individualize flow based on physiological parameters.	• Goal-directed perfusion targeting DO_2_i ≥ 280 mL/mi/m^2^ has been associated with lower lactate levels and improved postoperative markers, including creatinine, ventilation time, and ICU stay. [32]	SvO_2_ >65–70%
	• Prioritize DO_2_i over flow.		Lactate <2 mmol/L
			Adequate CO_2_ clearance (PaCO_2_ 35–45 mmHg)
			Hb >7 g/dL during CPB
Oxygenator selection	• Avoid routine oversizing based on actual BSA.	• Oversizing increases prime volume and hemodilution. (Blessing et al. [33]).	Preoperative LBM-derived flow estimate
	• Match oxygenator capacity to LBM-derived flow and expected gas exchange needs.		Expected CO_2_ production (200–250 mL/min/m^2^)
	• Be prepared for extra volume in the reservoir, prep blood bags or extra cardiotomy reservoir.		Monitor PaO_2_ and PaCO_2_, not oxygenator “capacity”
Anticoagulation strategy	• Avoid linear heparin dosing based on actual body weight.	• Lean or adjusted weight-based heparin dosing, followed by ACT-guided titration, is supported by studies evaluating lean body mass–based anticoagulation strategies [36, 37].	ACT target per institutional protocol (e.g., >480)
	• Use dosing guided by ACT and, where available, heparin concentration.		Heparin concentration (if available)
	• Adjust based on measured effect, not body size.		Recheck ACT after redosing
			Monitor closely during rewarming and separation from CPB
Temperature & rewarming management	• Avoid rapid rewarming and temperature overshoot (>37 °C).	• Metabolic demand rises disproportionately during rewarming while oxygen delivery may remain constrained. The rewarming phase carries the highest risk of supply–demand mismatch, particularly in obesity [32, 38].	Arterial outlet temperature ≤ 37 °C
	• Anticipate increased metabolic demand during rewarming.		Core + Bladder temperature (e.g., nasopharyngeal) 36.0–37.0 °C
	• Adjust flow, hemoglobin, and gas exchange before demand rises.		Monitor DO_2_i, SvO_2_, and lactate closely during rewarming
			Rewarm gradually (≤0.5–1.0 °C / 3–5 min)
Overall perfusion principle	• Adopt a physiology-driven, patient-specific approach.	• Obesity is a state of physiological mismatch; excess adipose tissue should not be treated as metabolically equivalent to lean tissue. Dynamic management improves outcomes and reduces complications (Blessing et al. [33]).	Trends over time rather than single values
	• Prioritize oxygen delivery, not weight-based targets.		Integrate clinical context, laboratory data, and perfusion metrics
	• Continuously reassess and adapt throughout CPB.		Team communication essential during all phases of CPB

The table summarizes key perfusion domains, including flow strategy, oxygenator selection, anticoagulation, temperature and rewarming management, and overall perfusion principles. Recommendations emphasize lean body mass–based estimation, dynamic oxygen delivery–guided perfusion, avoidance of unnecessary circuit oversizing, individualized anticoagulation, and proactive management during rewarming.

### Nursing considerations

Nursing care in morbidly obese cardiac surgical patients should be viewed as a risk prevention strategy rather than a purely logistical task. Existing evidence establishes that obesity increases the complexity of positioning, transfer, exposure, pressure area protection, and safe equipment use. The practical issue is that these risks begin before the patient enters the operating room; if equipment, staffing, table capacity, pressure-relieving surfaces, and positioning aids are not planned, preventable delays and injuries may occur. Patient-specific planning, including review of comorbidities, body habitus, mobility limitations, and positioning risk, allows the team to identify patients who require bariatric equipment, additional staff, transfer devices, table extensions, arm supports, and pressure redistribution surfaces [39]. Van Wicklin similarly emphasizes that inadequate equipment availability increases risk to both patients and staff, supporting the need for proactive preparation rather than reactive problem-solving on the day of surgery [5].

The key nursing challenge is balancing surgical exposure with patient safety. Obesity may require additional retraction, limb support, lifting, and repositioning to allow vascular access, line placement, skin preparation, cannulation, and sternal exposure. However, these same maneuvers increase the risk of pressure injuries, nerve injuries, falls, staff injuries, skin-fold contamination, and prolonged preparation time. Preoperative skin assessment, especially within skin folds and pressure-prone areas, is therefore not simply documentation; it establishes a baseline, identifies existing injury or moisture-associated damage, and guides intraoperative protection. Sacral foam dressing may reduce pressure injury risk when applied preoperatively and continued into postoperative care, reinforcing the need for continuity between OR and ICU nursing practice [40].

Transfer and positioning strategies should also be individualized. Air-assisted devices may reduce manual lifting and staff injury, while immediate safety belt application and multidisciplinary briefing can reduce fall risk and clarify the positioning plan. Spruce et al. describe positioning as a balance between optimal surgical access and patient safety, which is particularly relevant in morbid obesity because the same positioning required for exposure can compromise ventilation, venous return, pressure distribution, or nerve protection [21]. Thus, nurses function as patient advocates during positioning by identifying excessive pressure, unsafe limb position, inadequate padding, or prolonged tissue compression before injury occurs.

Obesity-specific preparation also affects operative efficiency and surgical feasibility. Longer instruments, deeper sternal blades, additional retractors, and modified exposure tools may be required for larger patients. These measures should not be interpreted as convenience items; they reduce repeated manipulation, improve exposure, and may limit tissue trauma and operative delay. Similarly, annual training in safe patient handling and coordinated lifting during skin preparation is important because staff fatigue and repeated manual repositioning can increase risk to both the patient and the operating team [41].

Wound prevention represents another area where nursing, surgical, and infection control practices overlap. Obesity is associated with adverse outcomes after cardiac surgery, and metabolic abnormalities such as dysregulated branched-chain amino acid catabolism have been linked with comorbidity burden, prolonged hospitalization, and unfavorable discharge outcomes [29]. In extreme obesity, resource requirements increase, including bariatric beds, reinforced operating tables, bed extensions, transfer systems, and prolonged ICU support; however, available evidence does not support delaying necessary cardiac surgery solely for weight loss [30]. This creates a practical tension: obesity increases perioperative risk, but risk reduction must usually occur through perioperative pathway optimization rather than postponement.

Sternal and conduit harvest wound prevention should therefore be planned from incision to postoperative recovery. Morbid obesity increases harvest site infection risk, and bilateral internal mammary artery harvesting is often avoided in patients with BMI >40 kg/m^2^. To preserve peristernal perfusion, with endoscopic vein or radial artery harvesting preferred in selected patients [15]. For sternal closure, rigid fixation may improve mechanical stability, and Liao et al. reported that adding a single xiphoid plate with wire cerclage improved stability and reduced analgesic requirements [16, 42]. Closure strategies that reduce dead space and maintain soft tissue approximation are therefore physiologically plausible in patients with obesity, although evidence remains heterogeneous.

Finally, infection prevention requires integrating antisepsis, antibiotic prophylaxis, wound care, and postoperative management. Dual alcohol-based antiseptics, careful skin preparation, weight-appropriate antibiotic prophylaxis with intraoperative redosing when indicated, and incisional negative pressure wound therapy in high-risk patients may all contribute to reducing wound complications. External chest support may provide additional stability, although patient size can limit use. Transfer systems and bariatric beds also remain important after the operation because postoperative movement, turning, and mobilization continue to influence wound tension, pressure injury risk, and staff safety [18].

Overall, existing literature establishes that nursing care in morbidly obese cardiac surgical patients is central to safe positioning, pressure injury prevention, exposure support, wound protection, and staff safety. What is newer is the shift from viewing these measures as isolated nursing tasks toward recognizing them as part of a coordinated perioperative risk reduction pathway. The practical implication is that patients with obesity should have an anticipatory OR and ICU nursing plan that includes equipment readiness, transfer strategy, skin assessment, pressure protection, exposure support, wound prevention, bariatric postoperative resources, and dignity-preserving care throughout the perioperative course.

### ICU/postoperative care considerations

Postoperative care in patients with obesity after cardiac surgery should be managed as a coordinated, phenotype-driven risk-reduction strategy rather than according to BMI alone. Although obesity is associated with increased postoperative morbidity and resource use, its relationship with mortality is inconsistent across studies, with some cohorts suggesting an obesity paradox for selected outcomes such as bleeding or early mortality [43–46]. These findings should not be generalized to morbid obesity, as larger studies also link obesity with respiratory failure, infection, renal dysfunction, and greater ICU resource use [43, 44, 46, 47]. Management should therefore focus on the dominant postoperative vulnerabilities: respiratory failure, acute kidney injury (AKI), atrial fibrillation (AF), infection, thromboembolism, and hemodynamic instability.

Respiratory failure is one of the most consistent postoperative concerns. Increasing BMI is associated with reduced lung volumes, increased oxygen consumption and carbon dioxide production, reduced compliance, increased airway resistance, and increased work of breathing, increasing the risk of atelectasis and extubation failure. [22, 24, 48] Ranucci et al. reported a 1.7 *x* fold increase in postoperative hypoxia risk per incremental BMI class [49]. Extubation should therefore be individualized rather than driven by routine fast-track pathways, considering respiratory mechanics, gas exchange, OSA or hypoventilation risk, hemodynamic stability, analgesic requirements, and readiness for CPAP or non-invasive ventilation. Early multidisciplinary involvement may reduce prolonged ventilation, reintubation, respiratory infection, ICU readmission, and delayed mobilization [2, 28].

Renal protection should begin intraoperatively and continue in the ICU. AKI occurs in 7.7%–28.1% of patients with obesity after cardiac surgery, with renal replacement therapy required in 1.4%–3% [50]. Mechanisms include diabetes, metabolic syndrome, obesity-related glomerulopathy, inflammation, oxidative stress, CPB-related ischemia-reperfusion injury, and postoperative hemodynamic instability [50]. ICU management should prioritize adequate perfusion pressure and oxygen delivery, avoidance of fluid overload and nephrotoxins, close monitoring of urine output and creatinine, and individualized vasopressor, diuretic, and fluid therapy.

Obesity also increases susceptibility to postoperative AF, particularly when OSA, hypoxemia, pulmonary hypertension, or RV strain coexist. Postoperative AF is associated with stroke, respiratory failure, and increased mortality after cardiac surgery [48, 51]. De Santo and Esquinas further highlight the relationship between OSA and postoperative AF risk [52]. Prevention should integrate respiratory optimization, treatment of sleep-disordered breathing, electrolyte correction, avoidance of excessive fluid loading, and early recognition of RV dysfunction or pulmonary hypertension.

Hemodynamic assessment may be less reliable in patients with obesity because non-invasive blood pressure can be inaccurate, transthoracic echocardiography may be technically limited, and clinical assessment of fluid responsiveness is often difficult. In selected unstable patients, invasive arterial pressure monitoring and transesophageal echocardiography may provide more reliable guidance. Fluid and vasopressor therapy should be titrated to physiological response rather than actual body weight, as excessive fluid loading may worsen pulmonary mechanics, RV strain, tissue edema, wound tension, and renal congestion [53].

Infection and thromboembolism prevention require particular attention. Higher BMI is associated with increased sternal and harvest-site wound complications, longer ICU stay, and greater resource use, driven by tissue depth, impaired exposure, longer conduit harvesting time, reduced tissue perfusion, larger dead space, poor glycemic control, and inadequate antibiotic dosing or redosing [16, 17, 41]. Weight-appropriate prophylaxis and intraoperative redosing are therefore important [17, 54]. Obesity also promotes inflammation, hypercoagulability, venous stasis, and reduced mobility, increasing susceptibility to DVT and pulmonary embolism, while diagnosis can be more difficult in severe obesity [55]. Management should include glycemic control, wound surveillance, line stewardship, respiratory hygiene, early mobilization, mechanical prophylaxis, weight-appropriate pharmacological thromboprophylaxis, and a low threshold for imaging when suspicion persists [17, 41, 53].

Finally, postoperative metabolic management should distinguish adiposity from lean-tissue demand. Although adipose tissue is metabolically active, its oxygen consumption is lower than that of skeletal muscle or myocardium; much of the increased systemic demand in obesity is indirect, arising from higher cardiac output, myocardial work, respiratory effort, and the need to support excess body mass [1, 4, 5, 53, 56]. This supports postoperative and perfusion strategies guided by oxygen delivery, lactate, respiratory work, hemodynamics, renal function, and end-organ perfusion rather than actual body weight alone.

Overall, ICU care in patients with obesity should be phenotype-driven and multidisciplinary, prioritizing respiratory support, renal protection, hemodynamic precision, infection prevention, thromboprophylaxis, arrhythmia surveillance, glycemic control, early mobilization, and coordinated escalation when recovery targets are not achieved.

Table 2 summarizes the major perioperative domains affected by obesity.

**Table 2:** Obesity-specific perioperative challenges and practical management strategies in cardiac surgery.

Perioperative domain	Obesity-specific challenge	Clinical implication	Practical management strategy
Preoperative assessment	Diabetes	Respiratory failure	Assess beyond BMI:
	OSA	AKI	Cardiopulmonary status
	Pulmonary hypertension	Arrhythmia	OSA
	Renal dysfunction	Wound complications	Renal risk
	Low reserve	Prolonged ICU stay	Glycemic control
			Team planning
Surgical exposure and access	Increased tissue depth	Longer surgery	Deeper retractors
	Poor visualization	Bleeding	Longer instruments
	Difficult conduit harvest	Technical difficulty	Modified exposure
			Ultrasound access
			Experienced assistance
Wound healing and infection prevention	Reduced perfusion	SSI	Meticulous closure
	Dead space	Sternal wound complications	Weight-adjusted antibiotics
	Poor Glycemic control	Delayed recovery	Glycemic control
			Consider negative-pressure therapy
Airway and ventilation	Reduced FRC	Difficult intubation	Ramped positioning
	OSA	Hypoxemia	Preoxygenation
	Hypoventilation	Delayed extubation	Difficult-airway plan
	Rapid desaturation	Reintubation	Lung-protective ventilation
			PEEP
			Planned respiratory support
Anesthetic pharmacology	Altered drug distribution and clearance	Overdose	Use ideal/adjusted/LBM dosing
		Delayed emergence	Titrate to effect
		Instability	
		Underdosing	
Cardiopulmonary bypass flow strategy	BSA-based flow may overestimate demand	Overperfusion	Guide flow by DO_2_
		Hemodilution	Hemoglobin
		Oversized circuit	Temperature
		Transfusion	Metabolic demand
			LBM
Oxygenator and circuit selection	Calculated flow may exceed true metabolic demand	Circuit oversizing	Match circuit to Patient demand.
		Inadequate gas exchange	Expected flow
			DO_2_
			Gas exchange
			Hematocrit
Anticoagulation and reversal	Total-body-weight dosing may overestimate need	Bleeding	Use lean/adjusted dosing initially,
		Protamine exposure	Then ACT-guided titration and individualized reversal
		Transfusion	
		Under-anticoagulation	
Positioning and nursing care	Limited mobility	Pressure injury	Prepare bariatric equipment
	Transfer difficulty	Nerve injury	Padding
	Pressure risk	Falls	Transfer devices
		Staff injury	Skin checks
		Delays	Dignity-preserving positioning
Postoperative respiratory care	Atelectasis	Prolonged Ventilation	Early respiratory therapy
	OSA	Hypoxemia	Head-up positioning
	Hypoventilation	Reintubation	Mobilization
	Increased work of breathing	ICU readmission	CPAP/NIV
			Analgesia
			Cautious fluids
Renal protection	Diabetes		Optimize Perfusion;
	Inflammation		DO_2_
	Altered reserve	AKI	Avoid fluid overload and nephrotoxins
	CPB injury	Renal replacement therapy	Monitor urine/creatinine
	Fluid overload		
Thomboembolism prevention	Prothrombotic state	DVT	Early mobilization
	Venous stasis	PE	Mechanical prophylaxis
	Low mobility	Delayed recovery	Weight-appropriate VTE prophylaxis
			Low threshold for imaging
Overall care pathway	BMI does not capture risk, reserve, or body composition	BMI-only risk stratification may mislead	Use multidisciplinary planning

This table summarizes key perioperative challenges, clinical implications, and practical management strategies across surgical, anesthetic, perfusion, nursing, and ICU domains.

## Clinical practice recommendations

The practical approach is to assess obesity as a phenotype rather than BMI alone, define risk before surgery, and align surgical, anesthetic, perfusion, nursing, and ICU plans before induction. Intraoperatively, teams should prioritize oxygen delivery, respiratory mechanics, hemodynamic stability, renal protection, anticoagulation monitoring, and early escalation when targets are not achieved. This approach should be operationalized through preoperative team planning, with surgical teams addressing exposure and access, anesthetic teams planning airway and ventilation strategy, perfusionists defining flow, oxygenator, anticoagulation, and rewarming targets, nursing teams preparing equipment and positioning plans, and ICU teams anticipating respiratory, renal, thromboembolic, and infection-related complications. The contribution of this review is not the identification of a single new intervention, but the integration of established obesity related risks into a multidisciplinary, physiology-driven framework. Existing literature describes the risks associated with obesity in cardiac surgery; this review translates those data into practical perioperative recommendations, with particular emphasis on phenotype-based risk assessment, lean body mass-informed perfusion, oxygen delivery-guided CPB management, individualized circuit selection, and coordinated team planning.

## Conclusion

Cardiac surgery in morbidly obese patients requires a coordinated, physiologically driven perioperative strategy to address obesity-specific anatomical and functional challenges. Tailored anesthetic, perfusion, and surgical approaches, supported by close multidisciplinary collaboration, are central to optimizing outcomes. Further research is needed to refine perioperative protocols, improve risk stratification beyond BMI, and clarify long-term cardiovascular outcomes following surgery in this population.

## Limitations

This review has several limitations. Owing to marked heterogeneity in study design, reported cardiopulmonary bypass strategies, and outcome definitions, a quantitative Results section summarizing component-level frequencies and pooled outcomes was not generated. Consequently, the findings are presented as a structured narrative synthesis rather than a formal meta-analytic summary. The available evidence is predominantly observational, with limited randomized data and substantial heterogeneity in BMI definitions, surgical procedures, CPB strategies, and reported outcomes. Several recommendations, particularly regarding LBM-based perfusion, oxygenator sizing, anticoagulation dosing, and temperature management, are therefore based on physiological rationale and expert synthesis rather than direct comparative trials in morbidly obese cardiac surgical patients. These limitations support an individualized, physiology-driven approach rather than rigid protocolization.

**Glossary of terms**
ACT: Activated Clotting Time
AF: Atrial Fibrillation
AKI: Acute Kidney Injury
AKICPB: Acute kidney injury after cardiopulmonary bypass
BMI: Body Mass Index
BSA: Body Surface Area
CABG: Coronary Artery Bypass Graft
CPAP: Continuous positive airway pressure
D0_2_i: Indexed Oxygen Delivery
DVT: Deep vein thrombosis
GLP-1: Glucagon-like peptide-1
ICU: Intensive care unit
LBM: Lean Body Mass
LV: Left Ventricular
OR: Operating room
OSA: Obstructive Sleep Apnea
PEEP: Positive End- Expiratory Pressure
RV: Right Ventricular
V0_2_
: Oxygen Consumption
VTE: Venous Thromboembolism

## Data Availability

The research data are available on request from the authors.
